# Ex Opthalmie Goitre

**Published:** 1861

**Authors:** R. C. Hamill


### "EX OPTHALMIC GOITRE."

REPORTED TO THE CHICAGO ACADEMY OF MEDICAL SCIENCES. MARCH, 1861.

By R. C. HAMILL, M. D.

I was called December 29th, 1861, to see Mrs. H—., a widow lady, in her 45th year, who was supposed by her friends to be in the last stages of consumption. She had been ill for the last eighteen months, and under the care at different times of several physicians, before she came to this city, Nov. 1st., 1860. For three weeks immediately preceding my call, she had been declining very rapidly. She was not confined to her room, however, and came down a flight of stairs to see me.

I found her pale and sallow, reduced in flesh, exceedingly nervous and suffering great dyspnœa. When seated, she was obliged to lean forward to breathe with any degree of comfort. Her cough was constant, short and cornvulsive and the heart's action was distinctly perceptible across the room. Her hands and feet were always warm, and the temperature of the skin appeared to be above the natural standard. She could not wear so much clothing as others, and was uncomfortably warm in a temperature suited to the other members of the family.—Had at no time suffered pain except in the abdomen. Had frequent night sweats, swelling of the feet and ankles, and diarrhœa, her bowels being moved as often as once in three or four hours, the evacuations uniformly of a light clay color, appetite never satisfied, her tongue small and red, the secretion of urine small. She expectorated during the latter part of the night and morning, from six to eight ounces of tenacious white sputa, pulse 120 per minute. On applying my ear to the chest, a tumultuous, irregular action was observed, but of such an indefined and confused character, that I was unable to arrive at a satisfactory conclusion as to the true nature. The respiratory murmur was totally inaudible, ronchus was heard, only, under the upper part of the sternum. She sleeps on the right side altogether, with her head bolstered up.

The history of the case as far as I learned it at my first visit is as follows:

About one year and a half since, without any premonition, she suddenly ceased to menstruate; concurrent with which diarrhoea set in and has continued without material change up to the present time. Her appetite has been "like that of a child, almost uncontrolable" during a greater portion of that period. Has not suffered from Leucorrhea, or in any way with disease of the reproductive system since the cessation of the menstrual functions. The diarrhoea, cough, and palpitation being the only causes of complaint. No remedies that she had ever tried had in any degree been attended with relief.

I failed to arrive at a satisfactory diagnosis, and made up my mind to prescribe for the over-working heart, hoping, if I could control its action, to relieve the dyspnœa dependent upon a congested state of the lungs that must of necessity exist, and as a probable sequence relieve the cough and expectoration. Accordingly I ordered veratrum viride ʒ ij—syrup of squills ℥ ij—acet morphine grs iV. One teaspoonful every six hours, with directions if nausea should occur to diminish the dose and lengthen the interval to eight hours.

I saw her again January 1st, 1861. The medicine had been well borne, and she had experienced considerable relief, especially at night. Did not cough so frequently, and had less difficulty in breething, pulse 95, treatment continued.

January 6th. She met me at the door with the exclamation, "Doctor my cough is gone and I have walked this cold evening around this block without coughing once." She had slept soundly all night and could lie upon either side with entire comfort, and had no dysprea for forty-eight hours. Her bowels continued the same and the same inordinate appetite. In further examination of her case to-day, my attention was directed to the remarkable prominence of her eyes, and on enquiry, learned that that fulness had suddenly come upon her, one night, whilst on a steam-boat excursion, abôut nine years since, and that about the same time the Thyroid began to enlarge. She had been troubled with palpitation some time before this. That she had been treated for the bronchitis with the various preparations of Iodine, internally and externally, without ever experiencing any relief. That she had been nervous ever since, but was much worse since the "change of life," and that about once in five or six weeks she had increased violence of the cough and dyspnœa which would last for a few days and then subside in part, each succeeding attack apparently leaving her worse than she was before.

The Thyroid gland was not much enlarged and had escaped my observation at the former visits. On examination I found both lobes of the gland elongated and indurated. The gland was crescent shaped, the cornua running up and burying themselves under the muscles of the neck, pressing firmly upon the subjacent tissues. The diagnosis was now clear. I had a case of "Ex ophthalmic Goitre." There was no throbbing of the gland, neither was there any marked fullness in the blood vessels of the head—nor pain in the head. Her pulse now 65 per minute, was still irregular; the nervous symptoms relieved. The state of the bowels remained the same. Auscultation and percussion revealed no organic lesion of the lungs. The veratrum viride was continued once or twice in twenty-four hours, with a view of maintaining the heart's action at its present status. Ext. Hyosciami ʒj, Nit. Silver grs X, sub. Nit. Bismuth ℈ji XL pills. One three times a day was ordered for the diarrhoea.

10th. The pills had procured some relief, the bowels being moved only four times in the last twenty-four hours, otherwise as at last visit. Treatment continued.

16th. Found her suffering from a severe form of Catarrh, which was very prevalent at this time in the city. Pulse 90, had taken the veratrum but once a day, bowels the same.—It is worthy of note here, that the occasional pains in the abdomen, of which she complains, ceased, upon discontinuing the use of a hair dressing, composed of lac sulphur and sugar of lead, showing the importance of knowing not only the regimen of a patient, but in some cases of being made acquainted with the mysteries of the toilet. The veratrum to be taken twice a day and the pills continued.

20th. Cough has been very troublesome, and the expectoration larger than ever before, loose, opaque and slightly tinged with green and easily thrown off, dyspnœa but slight, can sleep only on the right side, night sweats, pulse 70, no further improvement of the bowels. Ordered for the cough, syrup morphine ℥ ij. C. Tinct., Benzoin ℥ ij. One teaspoonful of the mixture every four hours. The pills and veratrum to be discontinued, and the following mixture to be taken instead: Strychnine grs. jss. dilute Sulph. Acid ʒ ij. Mint water ℥ ji.—One teaspoonful three times daily.

25th. Greatly improved in every particular. Coughs and expectorates but little, bowels moved but twice in the last twenty-four hours, pulse 60. The fulness of the eyes appeared to be less, and she thought they closed more easily. I could observe no change in the Thyroid. The strychnine mixture continued, with the addition of one grain of Sulphate of Iron to each dose. Cough mixture suspended.

Feb. 1st. She is entirely free from cough, has no difficulty of breathing, no night sweats, can sleep comfortably on her back or on either side, tongue natural, bowels relieved, needs more clothing and requires as warm a temperature as the other members of the family. The Thyoid is evidently less, and not so hard, and the eyes less prominent, pulse 60 per minute.—Treatment continued.

Feb. 10th. The improvement highly encouraging. The same treatment was continued up to the time of her departure from the city, or the 11th March, at which time she was able to undertake a journey of nearly one thousand miles to her home. The irregularity of the heart's action continued, but I was unable to satisfy myself as to the true nature of the malady, but fear that there is organic leison which will defy the best directed efforts of the Physician.

I was induced to try the strychnine in this case, from the success attending its use in three cases reported by Dr. Murney, in the Dublin Hospital Gazette, and republished in the January No. of Braithwaite, coupled with the highly beneficial results I had obtained in my own practice from that drug, in chronic derangements of the bowels. In the February No. of the London Lancet, C. Hanfield Jones reports two cases, the symptoms and treatment of which correspond in a remarkable degree with this case.

It will be observed that I obtained the same result with the veratrum viride and morphine that M. Trousseau, and the French practitioners derived from full doses of Digitalis Venesection and the cold douche. How much the treatment might have conspired to the permanent relief of the patient, I am unable to determine. The relief to the dyspnœa, cough and palpitation, on the eighth day was complete ; but the diarrhoea was in no degree relieved. I should most probably have continued the pills, and occasional use of the veratrum with the hope of ultimate relief, if it had not been for the attack of Catarrh, which I found her laboring under on the 16th January, and which in a great measure had re-established the symptoms that existed at my first visit, the diminished dyspnœa and the state of the pulse, (90 per minute), being the only modifications perceptible at that time. The veratrum viride and the pills were continued until the active stage of the catarrh had passed and resolution supervened, which latter state of the case I found on the 20th, at which time I ordered the strychnine mixture.

There was no change in the proptosis, or of the thyroid gland, from the 6th to the 20th of January that I could observe, showing that whilst the veratrum and morphine had over-ruled the tumultuous action of the heart, and relieved the impending train of distressing symptoms, they had not, in a degree to be appreciated, removed what *I presume* to believe will eventually be found to be the continuing cause, namely, the indurated state of the gland ; but left the patient liable to a recurrence of the malady, whenever the effects of the medicinal agent should have subsided, and exposure to an exciting cause should again occur. Venesection or derivative depletion in this case, would have been not only injudicious, in my judgment, but highly prejudicial, and calculated only, to deprive the patient of strength, when she was already in a state of great debility. Although she had a ravenous appetite and indulged it fully without distress to the stomach, yet the ingesta passed so rapidly through the alimentary canal that a sufficient amount of blood-making material was not absorbed to keep up the powers of the organism.

M. Trousseau advances the opinion that this peculiar disorder should be classed among the "Neuroses." He admits that anæmica is very generally found to co-exist with "Ex ophthalmic goitre" but says that it depends upon a peculiar state of the blood as an effect, not as a cause," and resulting in "a great measure from the general perturbation observed in the function of nutrition." A cause may be of such a character, either from its inherent nature or from long persistent action, as to produce effects that cannot be healed by its removal. In the early stages of diseased function, if the cause be taken away, we have every reason to expect that the healthy organism will be reinstated in accordance with a natural law of the animal economy, but if organic changes have been established we might have a secondary cause, in that effect, operating upon the succeeding links in the chain of physiological relations, which has powers for evil, and is really as causative as if there had been no antecedent pathological action. The order of sequence in the characteristic symptoms of this disease, according to M. Tousseau, constituting the rule, is first, Palpitation, second Ex opthalmos, and third Goitre, co-existent with which have vicious nutrition as an effect. An effect of what? The deduction is, evidently of deranged nervous action, as the treatment indicates. If the poverty or impurity of the blood depends upon the pathological state of the nervous system, it follows as a necessity that that abnormal state of the blood must furnish an unhealthy stimulus to the nerves in return, which to a greater or less extent must debase and pervert their healthy influence. Taking for granted that the nervous system is first in the train of causes and effects, the fluids will be brought a state correspondent to that nervous action, and bear an equivalent relation to that derangement, in the same manner that the two systems do to each other in their normal condition, acting reciprocally on each other perpetuating the disease. If the treatment be addressed, properly, to the nervous system we can overrule, in a great measure the pathological action, by subduing the capacity for, and the susceptibility to, the offending cause, and thus procure a degree of toleration which resembles health, and if there is simply functional derangement, we may initiate a state of the organism, that will favor healthy action and result, ultimately, in a perfect cure.

This disease, according to M. Tousseau is rarely completely cured. In all the cases that have come a second time under his observation, the thyroid was found to be indurated. This state of the gland itself, to my mind furnishes a sufficient cause for the return of the proptosis and palpitation. Its enlarged and hardened substances pressing upon the vagi nerves suggest, at once interference with sympathies that reach the heart, and the idea is by no means visionary that the first link in the chain of pathological effects may be traced to the gland, and the failure to get a complete cure by the French practitioners may be owing to the fact that they have not recognized the primary importance of this peculiar condition of the thyroid body.

As to the cause of this singular disease I will not attempt even a speculation. The suggestion of Dr. Murney that it has its origin in mental impressions, is plausble. His deduction that bronchocele is the result of impaired innervation, or perverted action of nervous functions, in his locality at least, and not dependent on climate or snow water, as has been alleged in other countries, is new to me, as well as his views in relation to the functions of the thyroid, which he says is "analogous to the spleen, first in a mechanical view acting as a diverticulum to the brain, as we know the spleen does to the stomach; and, secondly, in its pathology; enlargement and congestion of the spleen are often, in certain localities, the result of malaria; very often it is caused by affections of the mind, and the remedy for this affection of the spleen is either tonics or astringents, or both."

In relation to the treatment, I have further only to say that the results obtained from the veratrum and morphine, although prescribed before I had reached the diagnosis of the case, were in the right direction, and arrested the depleting process carried on through the lungs by quieting the action of the heart and arteries, and allaying the general nervous excitement, but failed to furnish any hystogenic powers to the nutrient system. The strychnine and subsequently the iron were prescribed, with the hope of meeting this feature of the case. The result furnishes all the comment necessary.

The cause of the proptosis is a subject of considerable speculative interest, not so much in a therapeutic point of view, as from the hidden nature of the facts. Fatty hypertrophy, œdema of the cellular membrane and sanguineous congestion have each had their advocates, but have each failed to receive any satisfactory support from post mortem observation. A more probable conclusion, to my mind, is that the same state of pausis, that gives rise to the palpitation, extends to the orbit, impairing the energy of the muscles within it. As evidence of this, we have a drooping condition of the upper eyelid, resembling Ptosis, dependent upon a relaxed or atonic state of the levator muscle which discharges its functions with apparent reluctance. If the other muscles of the orbit are in the same state, as we have good reason to infer from their relative functions and sympathies, then the deformity can at once be accounted for. If the restraining force exercised upon the eye by the muscles proper to it be impaired, the effect would be to let the globe fall forward, and this outward tendency would be strongly favored by the pressure of the arterial circulation. During the paroxysms, which, according to M. Trousseau, are essential characteristics of this malady, it is highly probable there may be sanguineous congestion, and that during their intensity there is pain and fullness in the head; but when the paroxysm subsides, the fullness is only in part relieved—the tension is taken off, but the ex-ophthalmia still exists. Further, in support of this view, the deformity, occasionally, occurs in a very short space of time and without the knowledge of the patient, the first intimation of its existence being conveyed by some member of the family who has observed the changes. That was the fact in relation to the case of Mrs. H. During the course of my attention to her, I observed no evidence of of congestion or of œdema. The conjunctiva lay in loose folds at its reflection from the globe of the eye lids, which would not have been the case if there had been œdema of the cellular strictures within the orbit, and there was no fullness of the vessels carrying red blood, either to the conjunctiva or the sclerotic coat, which would have been the fact if the vessels within the orbit had been in a state of great vascular turgescence. If it had been produced by sanguineous congestion adequate to such effects, the sudden fullness and pain in the orbit would have demanded the attention of the sufferer;—when, if paresis, from a remote cause, had produced such an effect the want of cognizance could readily be understood.—Myopia, another effect of this malady, in some cases, meets easy solution under this view. When the nerve filaments from the cerrical ganglia of the sympathetic, which animate the longitudinal fibres of the iris are subjected to pressure to such a degree as to interfere with their normal functions, dilation will be the result; which is one of the conditions of the eye upon which shortness of vision is supposed to depend.

Additional support is brought to this pathological view, from the results obtained from strychnia. The peculiar therapeutic action of that drug in certain forms of enervation, where there is no inflammatory action or organic leison of the nerve centres is well known. The benign effects obtained from its exhibition in this case, by Drs. Murney and Jones, furnish strong support to the propriety of the treatment and reflect an argument in support of the opinion that Proptosis is the result of paresis induced by pressure upon nerves distributed to and controlling the movements of the eye.

